# Immune mRNA Expression and Fecal Microbiome Composition Change Induced by Djulis (*Chenopodium formosanum* Koidz.) Supplementation in Aged Mice: A Pilot Study

**DOI:** 10.3390/medicina60091545

**Published:** 2024-09-20

**Authors:** Brian Harvey Avanceña Villanueva, Huai-Ying Huang, Yu-Chang Tyan, Pei-Ju Lin, Chang-Wei Li, Hoang Minh, Lemmuel L. Tayo, Kuo-Pin Chuang

**Affiliations:** 1 International Degree Program in Animal Vaccine Technology, International College, National Pingtung University of Science and Technology, Pingtung 912, Taiwan; j11285355@mail.npust.edu.tw (B.H.A.V.);; 2 Demin Veterinary Hospital, Kaohsiung 811, Taiwan; 3 Department of Pet Care and Grooming, Ta Jen University, Pingtung 912, Taiwan; 4 Department of Medical Imaging and Radiological Sciences, Kaohsiung Medical University, Kaohsiung 807, Taiwan; 5 Department of Medical Research, Kaohsiung Medical University Hospital, Kaohsiung 807, Taiwan; 6 Center for Cancer Research, Kaohsiung Medical University, Kaohsiung 807, Taiwan; 7 Center for Tropical Medicine and Infectious Disease Research, Kaohsiung Medical University, Kaohsiung 807, Taiwan; 8 Livestock Disease Control Center of Chiayi County, Chiayi 612, Taiwan; 9 Department of Veterinary Medicine, National Chiayi University, Chiayi 600, Taiwan; 10 AllBio Life Incorporation, Taichung 408, Taiwan; 11 Department of Anatomy and Histology, Faculty of Veterinary Medicine, Vietnam National University of Agriculture, Hanoi 100000, Vietnam; 12 School of Chemical, Biological, and Materials Engineering and Sciences, Mapúa University, Manila City 1002, Philippines; 13 School of Graduate Studies, Mapúa University, Manila City 1002, Philippines; 14 Department of Biology, School of Medicine and Health Sciences, Mapúa University, Makati City 1200, Philippines; 15 Graduate Institute of Animal Vaccine Technology, College of Veterinary Medicine, National Pingtung University of Science and Technology, Pingtung 912, Taiwan; 16 School of Medicine, Kaohsiung Medical University, Kaohsiung 807, Taiwan; 17 School of Dentistry, Kaohsiung Medical University, Kaohsiung 807, Taiwan; 18 Companion Animal Research Center, National Pingtung University of Science and Technology, Pingtung 912, Taiwan

**Keywords:** aging, lung inflammation, djulis (*C. formosanum*), cytokines, Toll-like receptors, gut microbiome, gut-lung axis

## Abstract

***Background and Objectives***: The aging process has always been associated with a higher susceptibility to chronic inflammatory lung diseases. Several studies have demonstrated the gut microbiome’s influence on the lungs through cross-talk or the gut–lungs axis maintaining nutrient-rich microenvironments. Taiwan djulis (*Chenopodium formosanum* Koidz.) provides antioxidant and anti-inflammatory characteristics that could modulate the gut microbiome. This could induce the gut–lung axis through microbial cross-talk, thus favoring the modulation of lung inflammation. ***Materials and Methods***: Here, we investigate the immune mRNA expression in the spleen, fecal microbiome composition, and hyperplasia of the bronchial epithelium in aged 2-year-old BALB/c mice after 60 days of supplementation of djulis. ***Results***: The pro-inflammatory cytokines IFN-γ, TNF-α, and IL-1β, T; cells CD4 and CD8; and TLRs TLR3, TLR4, TLR5, TLR7, TLR8, and TLR9 were reduced in their mRNA expression levels, while the anti-inflammatory cytokines IL-2, IL-4, and IL-10 were highly expressed in the *C. formosanum*-treated group. Interestingly, the fecal microbiome composition analysis indicated higher diversity in the *C. formosanum*-treated group and the presence of butyrate-producing bacteria that are beneficial in the gut microbiome. The histopathology showed reduced hyperplasia of the bronchial epithelium based on the degree of lesions. ***Conclusions***: Our findings suggest that Taiwan djulis can modulate the gut microbiome, leading to microbial cross-talk; reducing the mRNA expression of pro-inflammatory cytokines, T cells, and TLRs; and increasing anti-inflammatory cytokines in the spleen, as cytokines migrate in the lungs, preventing lung inflammation damage in aged mice or the gut–lung axis. Thus, Taiwan djulis could be considered a beneficial dietary component for the older adult population. The major limitation includes a lack of protein validation of cytokines and TLRs and quantification of the T cell population in the spleen as a marker of the gut–lung axis.

## 1. Introduction

Aging has been linked with elevated levels of circulating inflammatory cytokines, which significantly contribute to the increased susceptibility of the elderly to various diseases, including diabetes, heart disease, and chronic pulmonary conditions such as emphysema, bronchitis, and chronic obstructive pulmonary disease (COPD) [1,2,3]. This age-associated decline in the fidelity and efficiency of the immune system is often referred to as immunosenescence, while the systemic elevation of basal inflammatory cytokines from altered immune cell functionality, particularly within the myeloid lineage, is commonly referred to as inflammaging [1]. However, studies of monocytes or macrophages in vitro have yielded conflicting results, as some studies have demonstrated an impaired capacity of myeloid cells to produce inflammatory cytokines in old age [1,4], whereas others have shown enhanced secretion of pro-inflammatory cytokines [5,6]. These discrepancies are often attributed to factors such as the source of monocytes, derived or resident macrophages, as well as the specific stimuli employed [7]. The age-associated changes increased susceptibility to chronic inflammatory lung diseases due to impaired immune function and cytokine dysregulation contributing to this predisposition [6,7,8,9,10]. Previous reports have indicated that advanced age is a significant risk factor for morbidity and mortality associated with the infection of severe acute respiratory syndrome coronavirus-2 (SARS-CoV-2), the causative agent of coronavirus disease 2019 (COVID-19), due to elevated production of inflammatory cytokines in the lungs [11,12].

On the other hand, the gut microbiota has mutualistic interactions with the lungs, providing stable nutrient-rich microenvironments that could influence the immune responses and confer health benefits to the host [13,14,15]. Emerging studies reveal that gut microbiota and their metabolites influence pulmonary health by enhancing immune responses or through direct protection from pathobionts through microbial cross-talk linking the lungs and gut microbiota, also known as the gut–lung axis [16,17]. Dysbiosis of the gut microbiota has been implicated in the development of acute or chronic lung diseases, such as asthma, tuberculosis, and lung cancer [17,18,19]. The major mechanisms of the gut–lung axis mostly involve the spleen, as microbial cross-talk occurs between the gut lumen and the lymphatic or blood vessels [20,21,22,23,24]. Metabolites and antioxidants derived from the gut provide homeostasis in the spleen, regulating the lymphocytes and cytokine release, which migrates to alveoli, modulating and reducing the infiltration of pro-inflammatory cells and/or cytokines in the lungs [25,26].

Taiwan djulis, also known as red quinoa (*Chenopodium formosanum* Koidz.), a plant that is native to Taiwan and has been cultivated by the Aborigines for over 100 years, has gained attention for its potential health benefits [27]. Studies have shown that djulis has rich beneficial nutrient compounds such as polyphenols, which have excellent antioxidant properties [28,29,30]. Several studies have elucidated the anticancer, antioxidant, anti-aging, and anti-inflammatory characteristics of djulis due to its excellent antioxidant bioavailability and bioactivity [30,31,32,33,34,35,36]. In addition to its polyphenol content, djulis has also been investigated for its potential protective effects on liver injury and fibrosis. Lin et al. [37] found that phenolic acids or extracts from djulis exhibited protective effects against carbon tetrachloride-induced liver injury and fibrosis in animals. A study conducted by Souza et al. [38] has explored the effects of djulis seed extracts on declarative memory deficits induced by scopolamine in mice. Djulis hull crude extract has been reported to improve insulin sensitivity and blood glucose while modulating the gut microbiota [39,40]. Combining djulis with *Lactobacillus acidophilus* in rats inhibits colon carcinogenesis [41]. The protective effects of djulis have also been demonstrated in liver injury [42,43] and gastric ulcers [44]. The beneficial effect of djulis has long been well established in different experimental models [28,29,30,31,32,33,34,35,36,37,38,39,40,41,42,43,44,45,46,47,48,49,50,51,52,53,54,55]. Its influence on modulating the gut microbiome [39] and improving digestive health could open the door to reducing age-related inflammation, especially in the lungs, through microbial cross-talk or the gut–lung axis.

Here, we aim to investigate and utilize Taiwan djulis to improve the gut microbiome to improve the gut–lung axis. This study determines the long-term beneficial effect of having djulis in the diet, improving gut health, such as the induction of immune mRNA expressions of cytokines, T cells, and TLRs in the spleen through microbial cross-talk. Our study may serve as a primary indication of the favorable outcome of djulis intake in reducing the risk of age-related inflammation induced by pro-inflammatory cytokine dysregulation.

## 2. Materials and Methods

### 2.1. Preparation of Djulis (C. formosanum) Powder

Taiwan djulis seeds or red quinoa (*C. formosanum*) were provided by the Department of Plant Industry, National Pingtung University of Science and Technology (Pingtung, Taiwan). It was then dried in an oven (Gallenkamp, Cambridge, UK) at 60 °C for 9 h. The oven-dried Djulis seeds were pulverized through an osterizer blender (Hitachi, Tokyo, Japan), producing the djulis powder.

### 2.2. Experimental Aged Mice

This study used aged BALB/c mice (2 years old, weighing 27–29 g) purchased from the National Laboratory Animal Center (Taipei, Taiwan) and adhered to the ethical guidelines set forth by the National Pingtung University of Science and Technology Institutional Animal Care and Use Committee (NPUST-IACUC). The experimental protocols were approved by the NPUST-IACUC with license numbers NPUST-110-048 and NPUST-112-183. The mice were housed individually in clean cages with sufficient light and adequate ventilation. The housing conditions were maintained at standard levels, including a 12 h light/12 h dark cycle, a temperature of 22 ± 1 °C, and unrestricted access to food and water. Two groups of aged mice were formed, the *C. formosanum*-treated group and the control group, with six mice per group (*n* = 6). For the experimental intervention, the mice in the *C. formosanum* treatment group were orally administered 4.3 g/kg of djulis powder daily, dissolved in 200 μL distilled water, over 60 days, followed by about 1 mL distilled water to secure swallowing, while the control group maintained the regular diet for mice. At the end of the study period, the mice were humanely sacrificed, and lung sections were collected for subsequent histological assessment.

### 2.3. Histopathological Analysis of Aged Mice’s Lungs

After the mice were humanely euthanized, the lungs were carefully harvested and fixed in 10% phosphate-buffered formalin solution and embedded in paraffin. For the histopathological examination, 5 µm thick tissue samples were stained with hematoxylin-eosin. To acquire the images, the samples were examined under the Nikon Eclipse E-200 microscope connected to the Nikon DS-L2 camera (Nikon Instruments, Tokyo, Japan). A licensed veterinary professional from the National Laboratory Animal Center (NLAC, NARLabs, Taipei City, Taiwan) assessed and scored the lesions. The degree of lesions was graded from 1 to 5 depending on severity: 0 = not present; 1 = minimal (<1%); 2 = slight (1–25%); 3 = moderate (26–50%); 4 = moderately severe (51–75%); 5 = severe/high (76–100%) [56].

### 2.4. Synthesis of cDNA

Total RNA was extracted from a 0.1 g mouse spleen using a TRIzol reagent following the manufacturer’s protocol (Invitrogen, Taipei, Taiwan, 15-596-018). The extracted RNA (5 μg 132 per reaction) was reverse-transcribed using RevertAid (ThermoFisher, Waltham, MA, USA, K1691), 11 μL of the extracted RNA was mixed with 1 μL Oligo(dt)18 primer, and the reaction was carried out in a Blue-Ray Biotech Turbo Cycler (Blue-ray Biotech, Taipei, Taiwan) at 65 °C for 5 min. After the reaction, 4 µL of 5X reaction buffer, 2 µL of 10 mM dNTP mix, 1 µL of RiboLock RNase inhibitor, and 1 µL of RevertAid M-MuLV RT were added, and quantification was set for 60 min at 42 °C and at 70 °C for 5 min to terminate the reaction.

### 2.5. Real-Time PCR for the mRNA Expression of Cytokines, T Cells, and Toll-like Receptors

The mRNA expression of the cytokines TNF-α, IFN-γ, IL-1β, IL-2, IL-4, and IL10; T cells CD4 and CD8; and Toll-like receptors TLR3, TLR4, TLR5, TLR7, TLR8, and TLR9 were measured in this study using β-actin as an internal control. Real-time PCR was performed using the StepOnePlus™ Real-Time PCR System (Applied Biosystem, Foster City, CA, USA, 43-766-00) using FastSYBR^®^ Green Master Mix (ThermoFisher, Taipei, Taiwan, 4385612) following the manufacturer’s instructions, with a final volume of 20 µL. Primers used in this study are listed in Table 1. Thermal cycles were performed as follows: polymerase activation at 95 °C for 20 s, followed by 40 cycles of denaturation at 95 °C for 3 s, annealing at 60 °C for 15 s, and extension at 72 °C for 15 s. The mRNA expression levels for each gene were expressed as fold change using the 2^−(ΔΔCt)^.

### 2.6. Fecal Microbiome Analysis

Fecal pellets were collected 60 days after being treated with or without djulis powder. Fecal pellet samples underwent genomic DNA extraction, quality control, rDNA variable region amplification, library construction, and Illumina sequencing (AllBio, Taipei, Taiwan). The 16s rDNA amplicon sequencing includes the library construction using specific primers for the amplification of prokaryotic 16s rDNA (V3, V4), and the data analysis of the variable region for the identification of the composition and abundance of the prokaryotic organisms in the samples were performed following the proprietary workflow of GENEWIZ (AllBio, Taichung, Taiwan). Sequencing was performed using an Illumina MiSeq. Demultiplexed fastq files were imported into QIIME 2 [57], and sequence quality control feature table generation and the other subsequent analyses were performed. Operational Taxonomic Units (OTUs) were clustered using Deblur [58], and construction of a phylogenetic tree was accomplished by means of mafft-alignment. The OTU table was rarefied to 21,479 sequences per sample for alpha and beta diversity calculations. Weighted and unweighted UniFrac distances were calculated in QIIME 2 [57,59]. The taxonomic assignment was performed against the Greengenes2 version 2022.10, full-length sequences using the q2-feature-classifier plugin in QIIME 2. For the detection of differential taxa, the linear discriminant analysis effect size (LEfSe) was used, an algorithm for high-dimensional biomarker discovery and characterizing the genomic differences between two or more biological conditions.

### 2.7. Statistical Analysis

GraphPad Prism 10.1.2 (Graph Pad Software Inc., San Diego, CA, USA) was used to analyze all the data. Data were presented as mean ± standard deviation (SD) of different groups. The statistical significance between the control and *C. formosanum*-treated group was analyzed using two-way ANOVA. Statistical significance was defined as *p* value < 0.0001.

## 3. Results

### 3.1. Body Weight of Aged Mice

**Figure 1:**
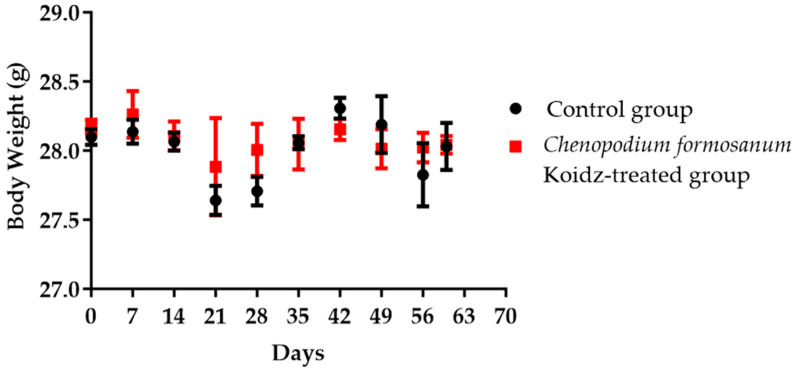
The aged mice’s body weight in the control group and *C. formosanum*-treated group were measured every 7 days. No significant change in body weight was observed with or without the *C. formosanum* treatment.

Aged 2-year-old BALB/c mice with 25–30 g body weight were chosen in this study. Body weight was monitored weekly and divided into non-treated as the control group and the *C. formosanum*-treated group. The *C. formosanum* treatment group did not change significantly in terms of body weight compared to the control group, as can be observed in Figure 1.

### 3.2. Cytokines and T Cell mRNA Levels in the Spleen after C. formosanum Treatment

**Figure 2:**
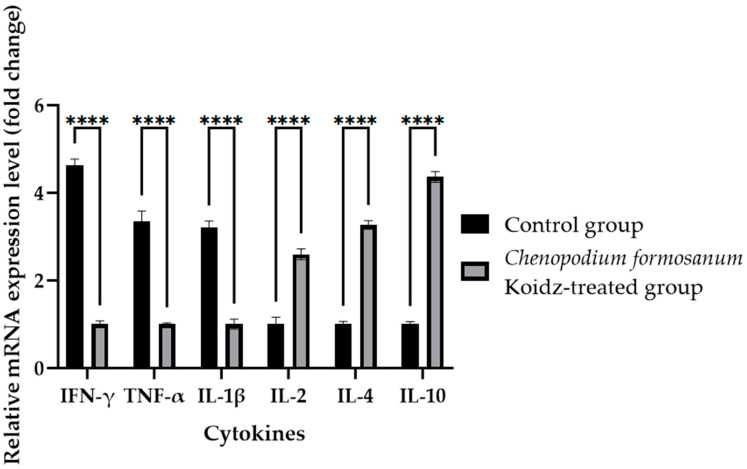
Cytokine fold change (2^−(ΔΔCt)^) levels in the aged mice. The *C. formosanum*-treated group had lower levels compared to the control group. The mRNA was extracted from the spleen of the aged mice after 60 days of treatment. Quantifications of the expression of each cytokine, IFN-γ, TNF-α, IL-1β, IL-2, IL-4, and IL-10, were performed using real-time RT-PCR, with β-actin as an internal control. Significant difference (*p* value < 0.0001) is notated as ****.

**Figure 3:**
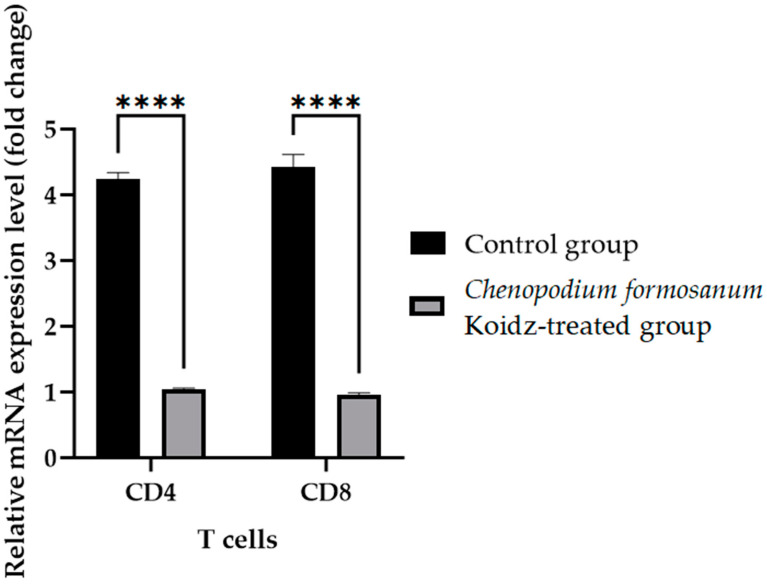
T cell fold change (2^−(ΔΔCt)^) levels in aged mice. The *C. formosanum*-treated group had lower levels compared to the control group. The mRNA was extracted from the spleen of the aged mice after 60 days of treatment. Quantifications of the expression of each T cell, CD4 and CD8, were performed using real-time RT-PCR, with β-actin as an internal control. Significant difference (*p* value < 0.0001) is notated as ****.

In our study, we investigated the effect of *C. formosanum* treatment on the expression levels of various cytokines and T cells as an immune marker in aged mice. The chronic inflammatory process observed in older adults, which serves as a marker for accelerated biological aging, is characterized by elevated levels of pro-inflammatory cytokines [5,6]. Following 60 days of treatment with *C. formosanum*, we observed a significant downregulation (*p* value < 0.0001) in the mRNA expression levels in the spleen of the pro-inflammatory cytokines interferon-gamma (IFN-γ), tumor necrosis factor-alpha (TNF-α), and interleukin-1β (IL-1β) and upregulation (*p* value < 0.0001) in the mRNA expression levels of the anti-inflammatory cytokines interleukin-2 (IL-2), interleukin-4 (IL-4), and interleukin (IL-10) in the *C. formosanum*-treated aged mice (Figure 2). Likewise, the mRNA expression levels of CD4 and CD8 T cells were lower in the *C. formosanum*-treated mice, as can be observed in Figure 3. These findings suggest that *C. formosanum* administration leads to a reduction in the expression of these pro-inflammatory cytokines.

### 3.3. Toll-like Receptor mRNA Levels in the Spleen after C. formosanum Treatment

**Figure 4:**
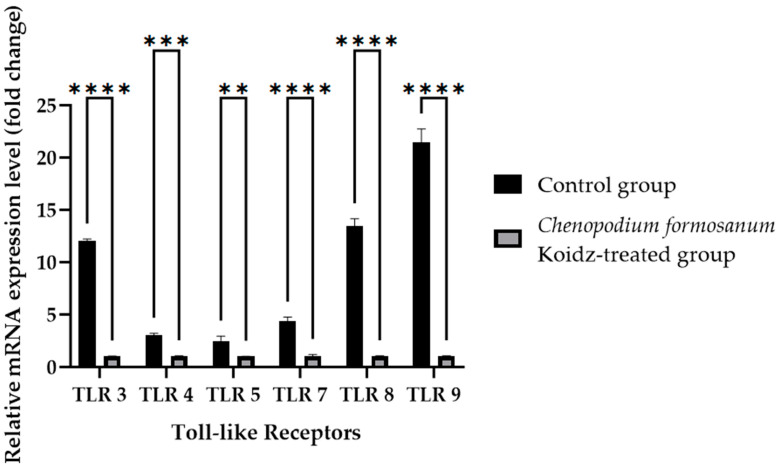
Toll-like receptor fold change (2^−(ΔΔCt)^) levels in the aged mice. The *C. formosanum*-treated group had lower levels compared to the control group. The mRNA was extracted from the spleen of the aged mice after 60 days of treatment. Quantifications of the expression of each Toll-like receptor, TLR3, TLR4, TLR5, TLR7, TLR8, and TLR9, were performed using real-time RT-PCR, with β-actin as an internal control. A significant difference (*p* value < 0.0001) is noted as ****, a *p* value of 0.0001 is noted as ***, and a *p* value of 0.0057 is noted as **.

Toll-like receptors (TLRs) are known to play crucial roles in immune responses and have been implicated in the aging process [60,61,62,63]. We investigated the potential influence of *C. formosanum* supplementation on the mRNA expression levels of TLRs. Real-time RT-PCR was performed of the harvested spleen tissues of aged mice who were sacrificed after 60 days of treatment. The results revealed a significant decrease in the mRNA expression levels of TLR3, TLR7, TLR8, TLR9 (*p* value < 0.0001), TLR4 (*p* value 0.0001), and TLR5 (*p* value 0.0057) after treatment with *C. formosanum,* as can be observed in Figure 4.

### 3.4. C. formosanum Treatment Reduced Hyperplasia of the Bronchial Epithelium

**Figure 5:**
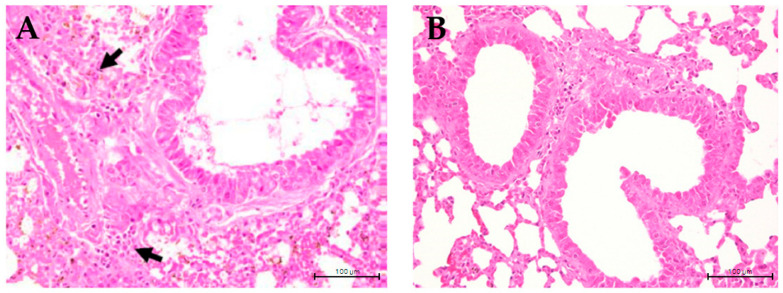
Histopathological image of aged mice lungs at ×400 magnification. (**A**) Lung section from non-treated aged mice showing hyperplasia of the bronchial epithelium (indicated with arrows, which are infiltrated inflammatory immune cells). (**B**) Lung section from *C. formosanum*-treated animals showing a standard histological architecture with no hyperplasia of the bronchial epithelium. The lung samples were stained with hematoxylin-eosin (H&E) stain and were observed under a light microscope.

Infiltration of inflammatory cells is considered a crucial indicator of the development of lung inflammation resulting in hyperplasia of the bronchial epithelium. The present study aimed to investigate the impact of *C. formosanum* on inflammation of the lungs, indicated by the hyperplasia formed in the bronchial epithelium (Figure 5). Following a 60-day control period, hyperplasia of the bronchial epithelium was observed in the control group (Figure 5A), while almost none was observed in the *C. formosanum*-treated group. However, administration of *C. formosanum* prevented the substantial elevation and infiltration of inflammatory cells on day 60 (Figure 5B). These findings suggest that *C. formosanum* holds anti-inflammatory effects in the lungs that are beneficial in aged mice. Furthermore, we provided a scoring matrix based on hyperplasia based on the degree of lesions of the bronchial epithelium, as can be observed in Table 2. The degree of lesions was graded from 1 to 5; again, the control group had a higher severity of lesions compared to the *C. formosanum*-treated group.

### 3.5. Administration of C. formosanum Enhanced Microbiota in Aged Mice

**Figure 6:**
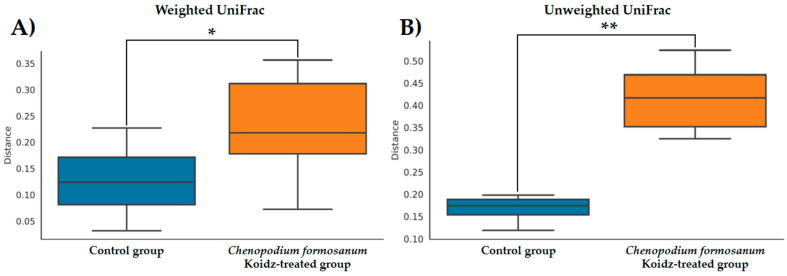
Boxplots of the (**A**) weighted and (**B**) unweighted UniFrac distances between the control (indicated by blue color box) and *C. formosanum*-treated aged (indicated by orange color box) mouse groups. A distance of 0 indicates that the samples are identical, and higher values (up to a maximum of 1) indicate the extent of differences between the compared models. Boxes show the 25th and 75th percentiles, with the median represented by a horizontal line. A significant difference is notated as * (*p* value < 0.05) and ** (*p* value < 0.001).

**Figure 7:**
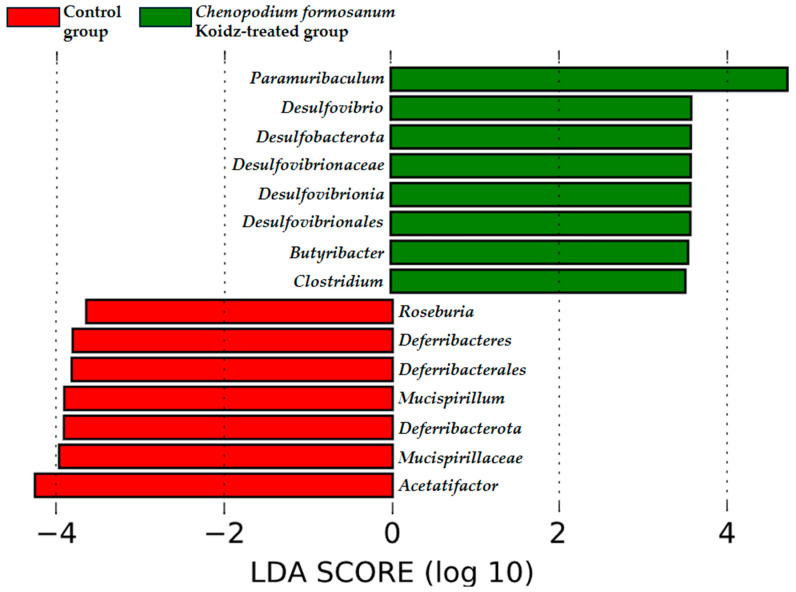
Characterization of microbiomes in the control and *C. formosanum*-treated aged mouse groups using LEfSe analysis. A histogram of the LDA scores (log10) computed for features with differential abundance. Positive (green) LDA scores implied an increased abundance of taxonomic features in the *C. formosanum*-treated aged mouse group, while negative (red) LDA scores signified the microbial biomarkers that were enriched in the control aged mouse group.

The community structure of the gut microbiota was analyzed using weighted and unweighted UniFrac distance matrices. The results, as depicted in Figure 6A (weighted) and Figure 6B (unweighted), clearly demonstrate a distinct separation between the two treatment groups. This indicates that the *C. formosanum* diet has a significant influence on the composition of the gut microbiota. The statistical analysis confirmed the significance of this differentiation, as indicated by the weighted (*p* < 0.05) and unweighted (*p* < 0.001) UniFrac metrics. We found significant differences in the community compositions between the two groups using LEfSe analysis. As shown in Figure 7, the microbial composition was also significantly different at the order level among groups. Next, we identified differentially abundant bacterial taxa between the control and djulis groups using LEfSe analysis. Eight bacteria taxa were significantly enriched in the *C. formosanum* treatment group: *Paramuribaculum*, *Desulfovibrio*, *Desulfobacterota*, *Desulfovibrionaceae*, *Desulfovibrionia*, *Desulfovibrionales*, *Butyribacter,* and *Clostridium* (Figure 7, green). Seven bacteria taxa were significantly enriched in the control group: *Roseburia*, *Deferribacteres*, *Deferribacterales*, *Mucispirillum*, *Deferribacterota*, *Mucispirillaceae*, and *Acetatifactor* (Figure 7, red).

## 4. Discussion

We conducted this experiment using 2-year-old BALB/c mice to mimic the aging process, pronounced inflammatory response, and attenuated lung inflammatory response [64,65,66,67]. On the other hand, djulis, *C. formosanum,* or red quinoa has been proven to have a beneficial influence on gut health and to modulate inflammation due to the presence of phenolic antioxidant compounds [28,29,30,31,32,37,38,45,46,50,53,68,69,70,71]. The experimental intervention used an additional 4.3 g/kg of *C. formosanum* powder with a daily intake for the aged mice or the *C. formosanum* treatment group, while the control group maintained the regular mice diet. This was to investigate the dietary implication of *C. formosanum* powder in aged mice as a supplementary diet while maintaining their regular diet.

Our study suggested that *C. formosanum* effectively suppresses inflammation in aged mice without causing any adverse effects, specifically in terms of the body weight (Figure 1). The beneficial effect of djulis in terms of managing body weight has been linked to the expression of genes that are responsible for regulating lipid metabolism and glucose breakdown [45,46,50,70]. Likewise, *C. formosanum* treatment enhances the production of anti-inflammatory cytokines, such as IL-2, IL-4, and IL-10, while decreasing pro-inflammatory cytokines, including IFN-γ, TNF-α, and IL-1β, based on the mRNA expression levels in the spleen (Figure 2). *C. formosanum* has been extensively studied for its polyphenolic content, mainly flavonoids, phenolic acids, and tannins, which are the three main types of polyphenols found in it and which exhibit potent antioxidant properties, as well as contributing to its anti-inflammatory and antitumor effects [5,30,31,32,37,38,53,68,69,70,71,72]. Furthermore, phenolic acids or extracts derived from djulis have been shown to protect animals from liver injury and fibrosis induced by carbon tetrachloride [53,70]. Based on these studies, it is suggested that both polyphenols and fibers that are present in *C. formosanum* may contribute to its prophylactic effects in aging, although the precise mechanisms require further investigation. *C. formosanum* may exert multiple functions, including anti-inflammatory effects, modulation of the colonic microorganism composition, and protection against immune cell infiltration in the lungs. Our research also shows that the mRNA expressions of IFN-γ, TNF-α, and IL-1β were reduced, but the IL-2, IL-4, and IL-10 mRNA expressions were increased after *C. formosanum* treatment in aged mice, but the detailed mechanism needs to be revealed in the future. Additionally, the mRNA levels of CD4, CD8, TLR-3, TLR-4, TLR-5, TLR-7, TLR-8, and TLR-9 in the spleen were observed to be reduced in the *C. formosanum* treatment group (Figure 3 and Figure 4). TLR4 has been linked with chronic low-grade inflammation and obesity in aged mice [73]. Overexpressions of TLR4 and TLR9 were reported to be related to liver degeneration related to aging-associated inflammation [74]. Toll-like receptors are crucial for the activation and regulation of different signaling pathways, and an aberrant or aggravated response of TLRs may lead to excessive inflammation, causing autoimmune disorders [60]. This clearly illustrates the immune mRNA induction of Taiwan djulis as a supplementary diet in the context of inflammaging. However, further research is required to elucidate the detailed mechanisms underlying these observations.

Despite these findings, our results are based on the mRNA levels of the spleen, which could represent post-transcriptional regulation and might not accurately represent actual protein concentrations. Further research using quantitative protein detection methods will be needed to confirm these findings on djulis supplementation at the protein level.

To the best of our knowledge, this is the first investigation into the effects of *C. formosanum* on the infiltration of inflammatory cells in the lungs of aged mice. Specifically, our results demonstrate that *C. formosanum* does not alter body weight and reduces the infiltration of inflammation cells into the lungs of aged mice based on the hyperplasia histopathology of the bronchial epithelium (Figure 5 and Table 2). The reduced hyperplasia lesion in the bronchial epithelium is the possible influence on the expression of IL-2, IL-4, and IL-10, which are well known as regulating cytokines favoring anti-inflammation activity [5,6]. Moreover, the fecal microbiome analysis revealed that *C. formosanum* treatment enhances the diversity of the gut microbiota compared to the control group. These results suggest that *C. formosanum* could have relevant anti-inflammatory effects that may be beneficial for aging individuals.

Previous studies have indicated that *C. formosanum* contains polysaccharides and phenolic compounds, which have the potential to modulate the gut microbiota [68,69]. In our present study, the analysis of weighted and unweighted UniFrac metrics suggested that *C. formosanum* possesses regulatory properties on the composition and diversity of the gut microbiota. As anticipated, *C. formosanum* treatment significantly increased the species richness and diversity indices (weighted and unweighted) of the gut microbiota, as can be observed in Figure 6. These results indicated that *C. formosanum* consumption promotes an increase in species richness and diversity, thereby exerting a positive effect on the maintenance of the gut microbiome.

The concept of the “gut–lung axis” has emerged as a link between the state of the gut microbiota and respiratory health outcomes [75,76]. The gut microbiota has been implicated in influencing lung health in various inflammatory diseases, including age-related respiratory conditions [23,75,77] and other inflammatory disorders [20,21,78,79,80]. Recent research has shown that the development of the intestinal and respiratory microbiota occurs simultaneously after birth [81], and there is continuous cross-talk between these two compartments, mediated by microorganisms through metabolic components that affect cytokine production. Microbial-mediated production of butyrate or short-chain fatty acids may promote anti-inflammatory responses by directly enhancing the production of IL-10 from regulatory cells [22,82,83] or by suppressing the expression of IL-12 p35, IL-12 p40, IL-1β [84], and TNF-α [85]. The LEfSe analysis indicated that *Butyribacter* and *Clostridium* (Figure 7) were rich in the *C. formosanum*-treated group, capable of synthesizing butyrate, which is beneficial for the gut–lung axis, and exhibiting potent anti-inflammatory effects [22,82,83,84,85,86]. Moreover, previous research indicates that aging is often associated with a reduction in the abundance of butyrate-producing bacteria and damage to the gut integrity [77]. The rich antioxidant properties of *C. formosanum* may contribute to improving gut health, specifically butyrate-producing bacteria, which leads to the expression or release of anti-inflammatory cytokines modulating the pro-inflammatory cytokines, T cells, and TLRs, and reducing hyperplasia of the lungs.

Djulis intake may provide bioactive compounds that could aid the gut microbiome in improving gut health, resulting in the necessary release of microbial metabolites [30,33,36,42,43,45,46,48]. Modulating the gut microbiome improves the gut lumen, allowing for microbial cross-talk between the gut lumen and the blood or lymphatic vessels [39,44]. This changes the immune mRNA expression in the spleen or the gut–spleen axis, regulating pro-inflammatory cytokine release and the production of anti-inflammatory cytokines [26]. Such cytokines migrate to the lungs, safeguarding the integrity and infiltration of inflammatory cells or reducing hyperplasia. They may migrate back to the gut and stimulate the gut–lung axis [22,23,25].

Our study provided initial insights into the beneficial effect of Taiwan djulis intake in aged mice. However, a major limitation of this study includes the lack of protein validation of cytokines and TLRs and of quantifying CD4 and CD8 T cell populations in the spleen as a marker for the gut–lung axis. Moreover, the validation of the cytokines and TLRs in the protein levels, accurately determining the CD4 and CD8 T cell population in the spleen, biochemical pathways influenced by djulis in the gut microbiome and microbial cross-talk, and the underlying mechanisms through which djulis exerts its effects, are other limitations of this study. Additionally, further investigations may examine the specific properties and underlying mechanisms through which Taiwan djulis exerts its effects and examine its potential therapeutic benefits in greater detail.

## 5. Conclusions

In conclusion, oral administration of djulis or *C. formosanum* effectively modulates the gut microbiome and reduces the mRNA expression of pro-inflammatory cytokines (IFN-γ, TNF-α, and IL-1β), T cells (CD4 and CD8), and Toll-like receptors (TLR-3, TLR-4, TLR-5, TLR-7, TLR-8, and TLR-9), while it enhances the mRNA expression of anti-inflammatory cytokines (IL-2, IL-4, and IL-10) in the spleen. Likewise, it preserves the lung architecture and reduces hyperplasia of the bronchial epithelium. A major limitation of this study includes the lack of protein validation in cytokines, TLRs, and T cells in the spleen as markers of the gut–lung axis. Additional investigations may be required for Taiwan djulis to determine its immunomodulatory effects and ability to induce the gut–lung axis. These findings hold potential implications for future pharmacological or dietary interventions targeting age-related conditions.

## Figures and Tables

**Table 1:** The sequence of specific oligonucleotide primers and optimal amplification conditions for mouse cytokines, T cells, and Toll-like receptor quantification using real-time RT-PCR.

Name	Type	Length	Primer Sequence	Product Size	Tm	Ref. Sequence
Mouse IL-1β	Forward	21	GAAATGCCACCTTTTGACAGTG	115 bp	60 °C	NM_008361.4
	Reverse	22	TGGATGCTCTCATCAGGACAG			
Mouse IL-2	Forward	23	TGAGCAGGATGGAGAATTACAGG	119 bp	60 °C	NM_008366.3
	Reverse	23	GTCCAAGTTCATCTTCTAGGCAC			
Mouse IL-4	Forward	20	CCATATCCACGGATGCGACA	254 bp	60 °C	NM_021283.2
	Reverse	20	AAGCCCGAAAGAGTCTCTGC			
Mouse IL-10	Forward	21	GCTCTTACTGACTGGCATGAG	104 bp	60 °C	NM_010548.2
	Reverse	20	CGCAGCTCTAGGAGCATGTG			
Mouse IFN-γ	Forward	20	ACTGTGATTGCGGGGTTGTA	198 bp	60 °C	NM_008337.4
	Reverse	20	ACATTCGAGTGCTGTCTGGC			
Mouse TNF-α	Forward	23	CCCTCACACTCAGATCATCTTCT	60 bp	60 °C	M13049.1
	Reverse	19	GCTACGACGTGGGCTACAG			
Mouse CD4	Forward	21	AGGTGATGGGACCTACCTCTC	102 bp	60 °C	NM_013488.3
	Reverse	20	GGGGCCACCACTTGAACTAC			
Mouse CD8	Forward	19	CCGTTGACCCGCTTTCTGT	120 bp	60 °C	NM_009857.1
	Reverse	21	CGGCGTCCATTTTCTTTGGAA			
Mouse β-actin	Forward	20	GGCTGTATTCCCCTCCATCG	153 bp	60 °C	NM_007393.5
	Reverse	22	CCAGTTGGTAACAATGCCATGT			
Mouse TLR3	Forward	22	GTGAGATACAACGTAGCTGACTG	161 bp	60 °C	AF355152.1 2
	Reverse	23	TCCTGCATCCAAGATAGCAAGT			
Mouse TLR4	Forward	20	AGCTCCTGACCTTGGTCTTG	266 bp	60 °C	NM_025817.4
	Reverse	20	CGCAGGGGAACTCAATGAGG			
Mouse TLR5	Forward	21	GCAGGATCATGGCATGTCAAC	129 bp	60 °C	NM_016928.4
	Reverse	21	ATCTGGGTGAGGTTACAGCCT			
Mouse TLR7	Forward	21	ATGTGGACACGGAAGAGACAA	207 bp	60 °C	AY035889.1 1
	Reverse	22	GGTAAGGGTAAGATTGGTGGTG			
Mouse TLR8	Forward	21	GAAAACATGCCCCCTCAGTCA	110 bp	60 °C	AY035890.11
	Reverse	23	CGTCACAAGGATAGCTTCTGGAA			
Mouse TLR9	Forward	21	ATGGTTCTCCGTCGAAGGACT	117 bp	60 °C	AF314224.1
	Reverse	19	GAGGCTTCAGCTCACAGGG			

This table shows the sequences of forward and reverse primers used in real-time RT-PCR reactions for cytokines and Toll-like receptor mRNA expressions. All the primers were synthesized by MDBio (MDBio, Taipei City, Taiwan).

**Table 2:** Scoring matrix of hyperplasia of the bronchial epithelium based on the degree of lesions.

Control Group		*Chenopodium formosanum* Koidz-Treated Group	
Aged Mouse	Degree of Lesions	Aged Mouse	Degree of Lesions
Control mouse 1	3	Treated mouse 1	1
Control mouse 2	3	Treated mouse 2	2
Control mouse 3	3	Treated mouse 3	1
Control mouse 4	2	Treated mouse 4	1
Control mouse 5	3	Treated mouse 5	1
Control mouse 6	3	Treated mouse 6	1

The degree of lesions was graded from 1 to 5 depending on severity: 0 = not present; 1 = minimal (<1%); 2 = slight (1–25%); 3 = moderate (26–50%); 4 = moderately severe (51–75%); 5 = severe/high (76–100%).

## Data Availability

All data generated and analyzed in this study are included in this article.
